# Whole Brain Approaches for Identification of Microstructural Abnormalities in Individual Patients: Comparison of Techniques Applied to Mild Traumatic Brain Injury

**DOI:** 10.1371/journal.pone.0059382

**Published:** 2013-03-26

**Authors:** Namhee Kim, Craig A. Branch, Mimi Kim, Michael L. Lipton

**Affiliations:** 1 The Gruss Magnetic Resonance Research Center, Albert Einstein College of Medicine of Yeshiva University, Bronx, New York, United States of America; 2 Department of Radiology, Albert Einstein College of Medicine of Yeshiva University, Bronx, New York, United States of America; 3 Department of Physiology and Biophysics, Albert Einstein College of Medicine of Yeshiva University, Bronx, New York, United States of America; 4 Department of Epidemiology and Population Health, Albert Einstein College of Medicine of Yeshiva University, Bronx, New York, United States of America; 5 Department of Psychiatry and Behavioral Sciences, Albert Einstein College of Medicine of Yeshiva University, Bronx, New York, United States of America; 6 The Dominick P. Purpura Department of Neuroscience, Albert Einstein College of Medicine of Yeshiva University, Bronx, New York, United States of America; 7 Department of Radiology, Montefiore Medical Center, Bronx, New York, United States of America; Cuban Neuroscience Center, Cuba

## Abstract

**Purpose:**

Group-wise analyses of DTI in mTBI have demonstrated evidence of traumatic axonal injury (TAI), associated with adverse clinical outcomes. Although mTBI is likely to have a unique spatial pattern in each patient, group analyses implicitly assume that location of injury will be the same across patients. The purpose of this study was to optimize and validate a procedure for analysis of DTI images acquired in individual patients, which could detect inter-individual differences and be applied in the clinical setting, where patients must be assessed as individuals.

**Materials and Methods:**

After informed consent and in compliance with HIPAA, 34 mTBI patients and 42 normal subjects underwent 3.0 Tesla DTI. Four voxelwise assessment methods (standard Z-score, “one vs. many” t-test, Family-Wise Error Rate control using pseudo t-distribution, EZ-MAP) for use in individual patients, were applied to each patient’s fractional anisotropy (FA) maps and tested for its ability to discriminate patients from controls. Receiver Operating Characteristic (ROC) analyses were used to define optimal thresholds (voxel-level significance and spatial extent) for reliable and robust detection of mTBI pathology.

**Results:**

ROC analyses showed EZ-MAP (specificity 71%, sensitivity 71%), “one vs. many” t-test and standard Z-score (sensitivity 65%, specificity 76% for both methods) resulted in a significant area under the curve (AUC) score for discriminating mTBI patients from controls in terms of the total number of abnormal white matter voxels detected while the FWER test was not significant. EZ-MAP is demonstrated to be robust to assumptions of Gaussian behavior and may serve as an alternative to methods that require strict Gaussian assumptions.

**Conclusion:**

EZ-MAP provides a robust approach for delineation of regional abnormal anisotropy in individual mTBI patients.

## Introduction

Measures of fractional anisotropy (FA) derived from Diffusion Tensor Imaging (DTI) reveal white matter abnormalities in mTBI, consistent with traumatic axonal injury (TAI), the presumptive pathologic substrate of adverse clinical outcomes after TBI (e.g.,[1]–[9]). Voxelwise analyses applied to mTBI research, almost universally compare groups of individuals. These studies thus implicitly assume that the spatial distribution of mTBI pathology will be the same across subjects, as only changes affecting a common location across the patient group will be identified as abnormal. This approach is inherently insensitive to intersubject variation in location of pathology. Since the spatial distribution of mTBI pathology among individual patients depends upon location and mechanism of injury, and given the wide variation in mechanism of injury and patient characteristics, this is a highly questionable assumption [10], [11]. Furthermore, clinical use of DTI requires assessment of individual patients. An approach to identifying loci of brain injury in individual mTBI patients is needed to fully understand the nature and extent of mTBI pathology toward personalizing and improving clinical practice.

Several studies have assessed DTI in individuals [4], [8], [12]–[14]. Viviani, et al. [12] applied a pseudo t-statistic with spatially smoothed standard deviation and degrees of freedom (DF) calibrated by cross-validation. They identified abnormal regions of the apparent diffusion coefficient (ADC) for single stroke and glioblastoma patients, with thresholds optimized for the Family-Wise Error Rate (FWER) based on the calibrated pseudo t-distribution. In many neuroimaging studies focused on lesion detection, spatial smoothing has not been carried out due to the risk for blurring of lesion margins. However, the FWER for control of Type-I errors in neuroimaging data may be overly conservative, especially when the images are not smoothed sufficiently [15]. The “one vs. many” T-test approach, employing a priori thresholds (individual voxel and cluster level) has been previously applied to mTBI patients [4], [8], [13] and the standard Z-score approach [14], [16]. However, these authors did not report validation or effectiveness testing of their thresholds.

This study aims to validate “Enhanced Z-score Microstructural Assessment of Pathology” (EZ-MAP) described by Lipton et al. [17], for detection of regional FA abnormalities in individual mTBI patients, and to compare EZ-MAP to previously reported methods. Like other studies [4], [8], [12]–[14], [16], EZ-MAP compares a patient’s FA value to those from a normal reference group at each voxel. Therefore, assessment of abnormality for each voxel entirely depends on summary statistics, i.e., mean and standard deviation, from the chosen reference group. It follows that final results may vary with the composition of the reference group, with potential for highly unreliable inferences when the reference group is small as it was in previous studies (10–11 subjects in the reference groups reported by [4], [8], [13], [14]). We employed a bootstrap procedure to overcome the potential for sample-to-sample variation of Z-scores. We also address limitations of all the prior approaches including EZ-MAP and perform specific validation addressing robustness, sensitivity, specificity and diagnostic utility.

## Materials and Methods

### Ethics Statement

After Albert Einstein College of Medicine Institutional Review Board (IRB) approval, Health Insurance Portability and Accountability Act (HIPAA) compliance and written informed consent, subjects were prospectively enrolled, distinct from clinical care. Thirty-four mTBI patients from one hospital emergency department met inclusion/exclusion criteria (Table 1) and were enrolled between August 2006 and May 2010. Forty-two control subjects with no history of head injury were recruited through advertisements.

**Table 1:** Inclusion and exclusion criteria for patients.

Inclusion Criteria	Exclusion Criteria
18–67 years of age	Prior head injury
Emergency department diagnosis of concussion within 2 weeks	Hospitalization due to the injury
GCS = 13–15	Neurodevelopmental or neurological disorder
LOC <20 minutes	
Posttraumatic amnesia <24 hours	Major psychiatric disorder
No focal neurologic deficit	Illicit drug use within 30 days
English or Spanish proficiency	Skull fracture or abnormal CT

Note- Normal control subjects (age range: 18–67 years) met the same exclusion criteria as patients. CT = Computerized Tomography.

### Data Acquisition

Imaging was performed at 3.0-T (Achieva; Philips Medical Systems, Best, the Netherlands) using an eight-channel head coil (Sense Head Coil; Philips Medical Systems). T1-weighted whole-head structural imaging was performed using sagittal three-dimensional magnetization-prepared rapid acquisition gradient echo (MP-RAGE; 9.9/4.6; field of view, 240 mm; matrix, 240×240; and section thickness, 1 mm). T2-weighted whole-head imaging was performed using axial two-dimensional turbo spin-echo (4000/100; field of view, 240 mm; matrix, 384×512; and section thickness, 4.5 mm) and axial two-dimensional fluid-attenuated inversion recovery turbo spin-echo (1100/120; inversion time, 2800 msec; field of view, 240 mm; matrix, 384×512; section thickness, 4.5 mm; and average number of signals acquired, one) imaging. DTI was performed using single-shot echo-planar imaging (3800/88; field of view, 240 mm; matrix, 112×89; section thickness, 4.5 mm; independent diffusion sensitizing directions, 32; and *b = *1000 sec/mm^2^).

### DTI Preprocessing

The American Board of Radiology certified neuroradiologist reviewed MR images of all subjects (patients and controls) in random sequence, blind to clinical information and group membership (patient or control). The 33 diffusion-weighted image sets (32 diffusion sensitizing directions and the *b = *0 sec/mm^2^ image) were corrected for head motion and eddy current effects using an affine registration algorithm. FA was derived from DTI at each voxel using the FMRIB Diffusion Toolbox [18]. Preprocessing procedures implemented for DTI included skull stripping, echo-planar imaging distortion correction, intermediate rigid-body registration, registration to standard space, transformation of DTI to standard space, and white matter segmentation, in sequence. Non-brain voxels were removed from the MP-RAGE and turbo spin-echo images using FMRIB-FSL software [19]. Each brain volume was inspected section-by-section, and residual non-brain voxels were removed manually. Turbo spin-echo images were acquired with the same section thickness, position and orientation as DTI. Distortion correction was accomplished using a nonlinear deformation algorithm to match each echo-planar image to the corresponding turbo spin-echo volumes [20]. For intermediate rigid-body registration, each subject’s turbo spin-echo images were registered to their three-dimensional MP-RAGE volume using the Automated Registration Toolbox [21] three-dimensional rigid-body approach [22]. For registration to standard space, the nonlinear registration module in ART was used to register each subject’s three-dimensional MP-RAGE volume to a standard T1-weighted template (Montreal Neurological Institute atlas; MNI) [23]. For transformation of DTI to standard space, distortion correction, intermediate rigid-body registration, and standard space registration were applied to the calculated FA maps in a single resectioning operation using ART. Final cubic voxel size was 1 mm^3^, masked to exclude non-brain voxels from the analysis. For white matter segmentation, the fast automated segmentation tool in the FMRIB-FSL package [19] was used to generate a white matter mask for the three-dimensional MP-RAGE template brain images and restrict subsequent statistical analysis of FA to white matter voxels.

### Adjustment for Demographic Covariate Effects

Because application of our methods to clinical settings will require use of ready control data to assess a new patient, we chose not to match controls one-to-one with patients. However, controls were chosen with an even distribution of age, gender and educational attainment that fully brackets the range of the patients; no patient age or educational attainment exceeds all controls at either extreme. For the purpose of our validation experiments, we subdivided the control group into two similar subgroups of 21 controls each. We adjusted for the potential effects of age, gender and education using a linear regression model estimated from one of the subgroups (the reference group). FA images used in subsequent analyses were first adjusted by applying regression coefficients to voxels where effects were significant. Regression coefficients thus determined were applied to FA images of the remaining 21 control subjects (“normal control subjects”) and patients, but only at locations where effects on individual voxels were significant at p<0.05 and where more than 100 significant voxels formed a contiguous cluster. This approach was taken because application of the regression model to all regions will only add noise to the Gaussian Random Field (GRF), diminishing sensitivity [24].

### EZ-MAP

**Figure 1:**
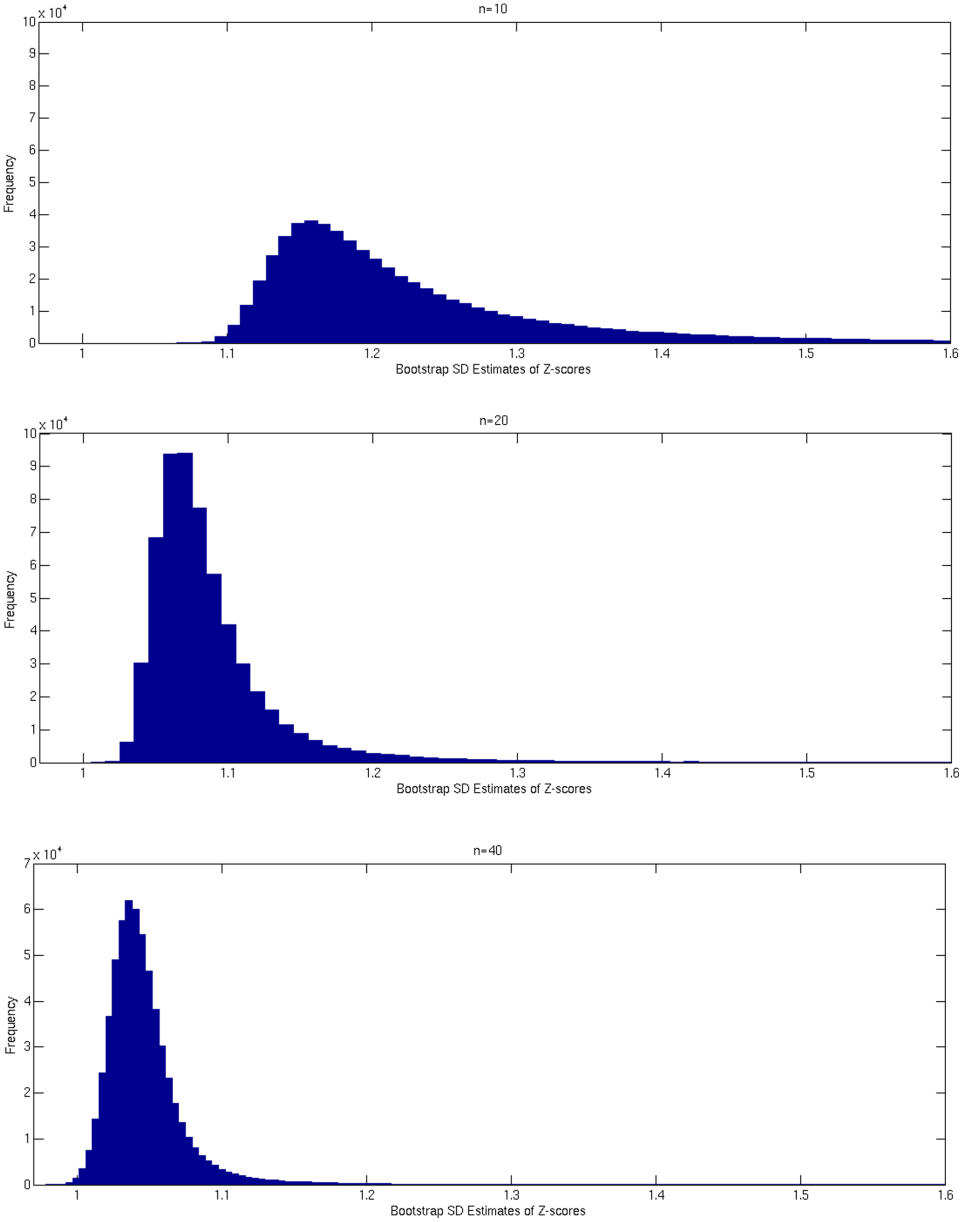
Histogram of bootstrap SD estimates. Each histogram of bootstrap SD estimates, 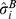, of Z-scores from all white matter voxels is based on a reference control group with size 10 (top), 20 (middle), and 40 (bottom), and produced by the procedure described in Text S1.

**Figure 2:**
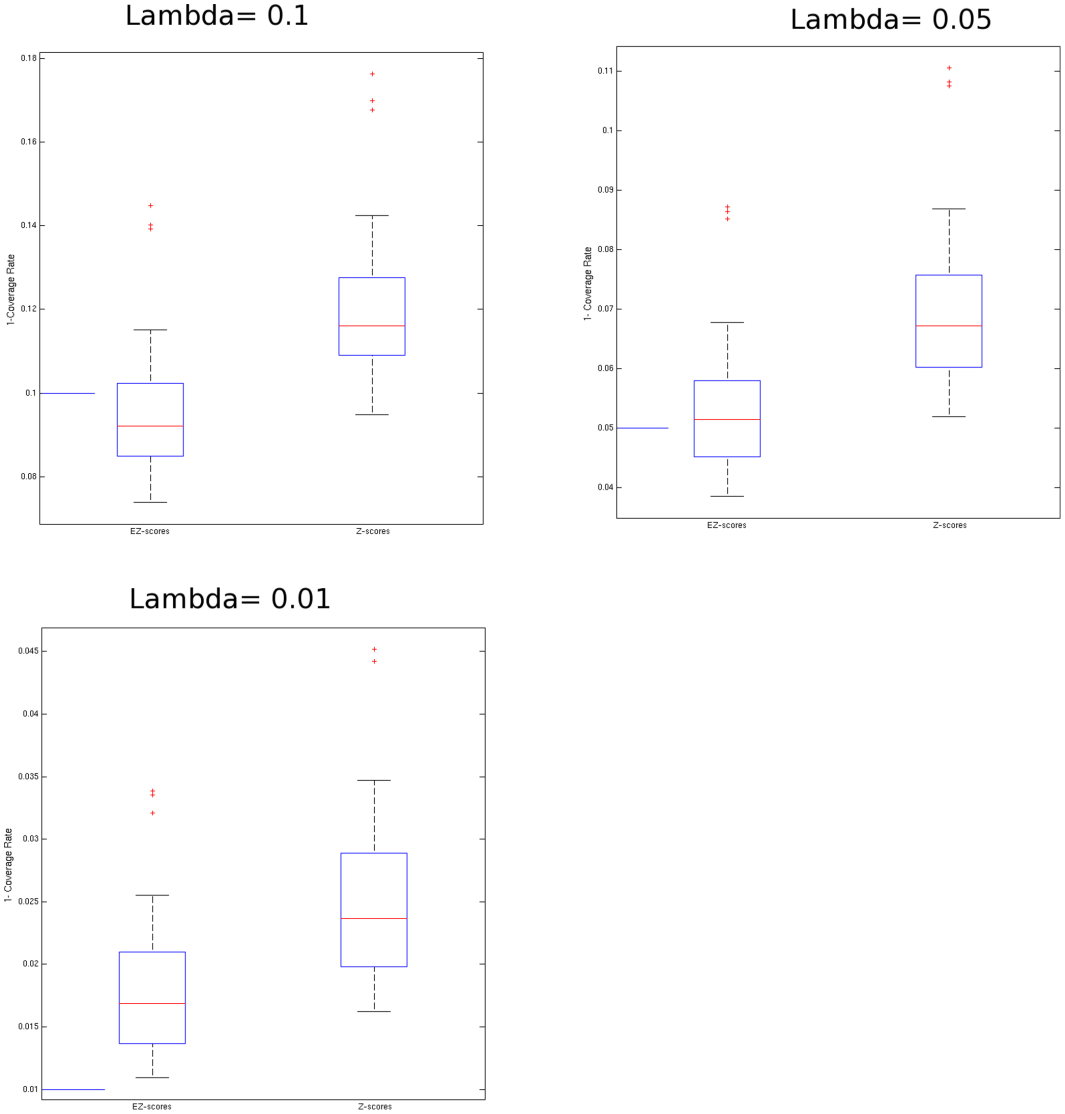
Box plots of uncoverage rates for normal control test subjects. Using 21 control subjects as the reference group, EZ-scores were derived for each normal control test subject (“Normal Subjects”, n = 21; different subjects than those comprising the reference group). The actual uncoverage rate, defined as the proportion of voxels lying outside of ± z_1-λ/2_ in each “Normal Subject”, was determined at three percentiles, corresponding to three theoretical uncoverage rates, (λ), 0.1, 0.05, 0.01. Box plots show the range of actual uncoverage rates across the 21 “Normal Subjects” for each of the three theoretical percentiles, each corresponding to the chosen λ. In each plot, closeness to λ, marked in blue, indicates better correspondence of the subject measurement to the expected distribution. Outliers in each box plot, marked with red+signs, are defined as values that are more than 1.5 times the interquartile range away from the top or bottom of the box. Observed uncoverage rates produced using EZ-score, as compared to the standard Z-score approach, indicate that EZ-score better represents the standard normal distribution and, therefore, provides a more accurate threshold for determination of abnormality with respect to a relatively small reference group of control subjects.

We computed the Z-score defined by 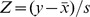 with 
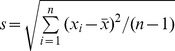
 at each voxel within a subject’s FA volume with reference values (mean and Standard Deviation (SD)) computed from the reference control group (n = 20), where one subject of the reference group in Table 2 was excluded for this calculation of mean and SD. Since the bootstrap procedure described in Text S1 requires n+1 control subjects to simulate Z-scores, each of which is a realization of deviation of a control subject’s FA from n control reference subjects, the reference group (Table 2) comprises 21 subjects. For each bootstrap implementation, (n+1) subjects were resampled, where for each resampling, first n subjects were grouped as a reference control group and the (n+1)th subject served as the control test subject. Ideally, although practically impossible, the reference mean and SD used to compute any Z-score should be derived from a very large control group. In our case, the ideal reference population would include all normal subjects within the demographic parameters defined above; we extracted subsets of this population as our control sample. In practice, the limited size of a control group (e.g., 10–11 subjects as previously reported [4], [8]), relative to the size of the entire reference population may mean that the control group does not optimally represent the full population from which it was selected. Therefore, the control group mean and SD may change with the composition of the selected control group, causing a bias (away from zero) and adding variance to the Z-score. Since the control group mean converges to the population mean at a faster rate than SD, additional variance is likely to be the most important factor contributing to variation of Z-scores across different control subgroups. Inferences based on Z-scores computed using only the control group SD might thus yield an unacceptably high rate of false positive results. Uniform application of higher Z-score thresholds to all voxels can be adopted in an effort to minimize false positive results, but may result in decreased detection power. We account for the potential excess variance in the Z-score at each voxel nonparametrically by employing a bootstrap procedure as described in Text S1. We term the Z-score based on this bootstrap-adjusted variance the Enhanced Z-score (EZ-score). The EZ-score at voxel (i) is then given in Equation (1).
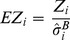
(1)where 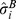 is the bootstrap SD estimate of Z-scores at voxel i from the bootstrap procedure (Text S1). The estimated variance from the bootstrap procedure for estimation of sample-to-sample variation of Z-scores may be greater than 1 (Figure 1). Therefore, determining abnormalities based on the EZ-score in Equation (1) will be more conservative than the assessment with the standard Z-score. The EZ-score approach adjusts each Z-score, with its potential variability induced by differing the reference group, and produces more robust results. This approach may provide a better coverage rate for FA values from new normal control subjects who were not part of the control group used for estimation of the mean and SD, in that the coverage rate is defined by the proportion of voxels lying between ±z_1-λ/2_, with a target coverage rate (1-λ)×100(%). This result is demonstrated in Figure 2. For all three coverage rates tested (90%, 95%, 99%). Coverage rates for the EZ approach are closer to target rates than those for the standard Z-score approach.

**Table 2:** Distribution of demographic variables across mTBI patients and control subjects.

		Total	Control Group 1(Reference)	Control Group 2(Test Subjects)	Patients
**Total**	**N**	**42**	**21**	**21**	**34**
**Age**	19–29	11	5	6	13
	30–39	12	6	6	13
	40–49	8	4	4	5
	50–59	9	4	5	–
	60+	2	2	–	3
	Min	20	20	21	19
	Max	67	67	59	64
	Mean	38.3	38.4	38.2	34.9
**Education**	<10	1	1	–	5
	11–13	10	4	6	12
	14–17	16	8	8	14
	18–20	7	4	3	3
	21–23	4	2	2	–
	24+	4	2	2	–
	Min	7	7	12	8
	Max	26	26	24	19
	Mean	16.6	16.5	16.7	13.6
**Gender**	Female	20	11	9	19
	Male	22	10	12	15

Potential variability induced by differing reference groups, which was estimated by the bootstrap SD procedure, was maximal with a smaller reference group and decreased as the number of reference subjects increased (Figure 1 (top: n = 10, middle: n = 20, bottom: n = 40)). Determination of an abnormality with standard Z-scores based on a small control group may therefore include a substantial number of false discoveries because sample-to-sample variation in FA among the normal control subjects is not accounted for.

We initially determined significance by assessing the Tail Probability at a voxel *i* (TP_i_) of the EZ-score from the standard Gaussian distribution (Equation (2)).
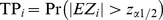
(2)


We applied two levels of thresholding to identify significantly abnormal voxels. First, each voxel must meet a threshold (α_1_) for the TP_i_ (Equation (2)) in order to be classified as abnormal. Second, the subset of these voxels that forms contiguous clusters meeting a size threshold (α_2_) is ultimately classified as abnormal. We determined the threshold for cluster size using GRF theory [24], which determines the significance of each suprathreshold cluster, a set of contiguous voxels which meet the individual voxel threshold (α_1_), and tested thresholds both uncorrected and corrected for multiple comparisons.

Receiver operating characteristic (ROC) analysis is suited to the assessment of complex diagnostic methods, such as neuroimaging, where theoretical validation of all aspects of a diagnostic procedure may be impossible. ROC is particularly useful in our case because it allows simultaneous assessment of multiple threshold values, α_1_ and α_2_, which may have a complex relationship to classification power.

ROC analysis requires explicit definition of true positive and false positive states. In our sample, however, no observable structural brain abnormalities were present on which to base a decision as to the presence of mTBI pathology. Furthermore, we expect that microstructural pathology will be present even in the absence of overt imaging abnormalities. Thus, we used ROC analysis to test the utility of EZ-MAP for classification of subjects as patients or normals in terms of the number of abnormal voxels detected. EZ-MAPs were generated for both 34 mTBI patients and 21 “normal control subjects”. We used a separate unique subgroup of normal subjects (not members of the “normal control group” tested [as mentioned at the end of the previous sentence]) as the “reference group” for computation of the EZ-MAP in each patient or “normal control subject”. That is, none of the “normal control subjects” for whom we computed EZ-MAPs were members of the “reference group” used to provide mean and SD for computation of the EZ-MAPs. The sole role of the “normal control group” in this study was to serve as test subjects or “pseudo-patients”. Using a range of combinations of the two thresholds (α_1_ and α_2_), ROC analysis identified optimal levels of the two thresholds, where AUC was maximal.

### One vs. Many t-test

The “one vs. many” t-test [4], [8] utilizes the t-distribution with n-1 (n = size of the reference group) DF. Individual voxels are classified as abnormal based on the t-score defined by
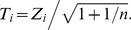
(3)


**Figure 3:**
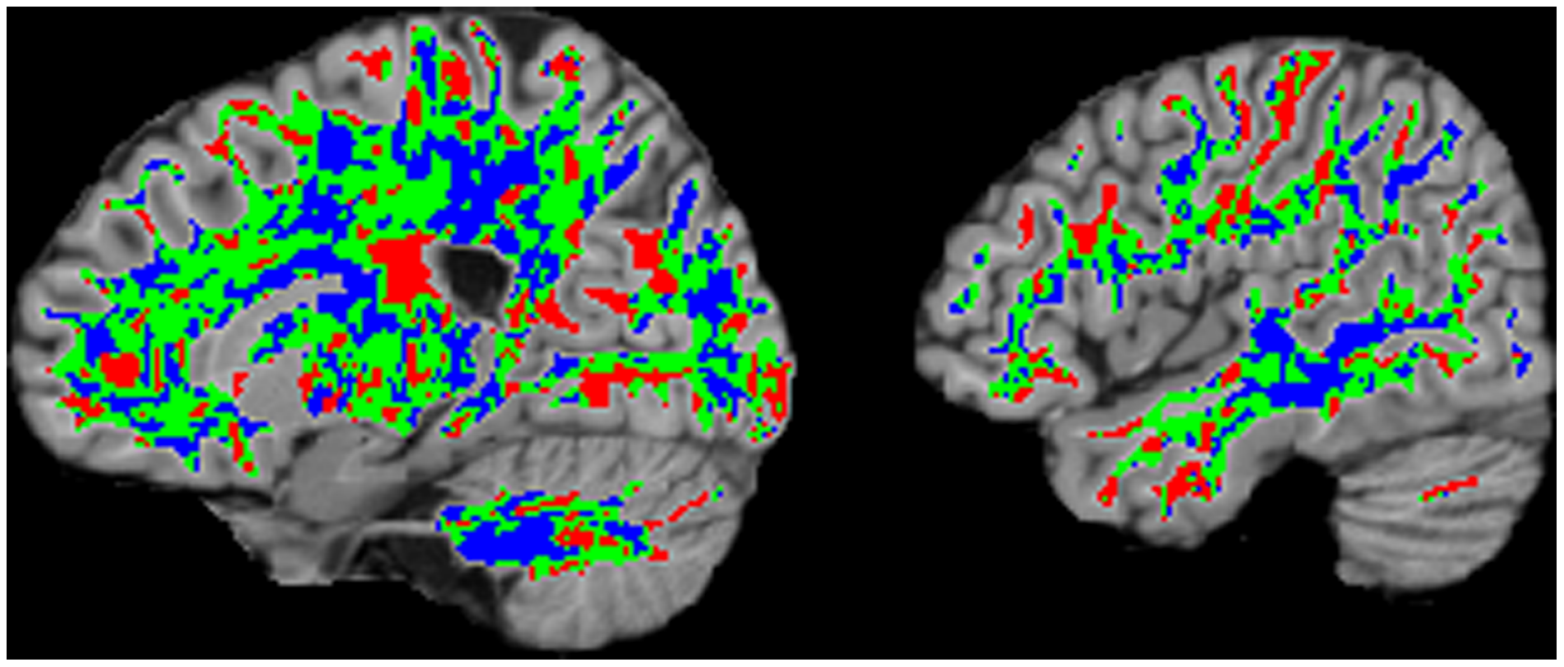
Spatial distribution of bootstrap estimated SDs. Voxelwise bootstrap estimated SDs are classified into 3 groups: under-dispersion, over-dispersion and close to the theoretical SD value. Voxels are classified as over- or under- dispersion if the estimated across-subject variation by the bootstrap procedure (Text S1) is substantially greater than the SD of the theoretical t distribution 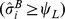 or substantially less than the SD of the theoretical t distribution 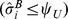, respectively, while voxels are classified as “close to the theoretical SD” of the t distribution if they are within ψ_L_ and ψ_U_, where ψ_L_ and ψ_U_ are determined by the SD of the t-distribution with the DF from the theoretical t distribution −/+10, respectively. Each class was colored coded: red (over-dispersion), blue (under-dispersion), and green (close to the theoretical SD) and superimposed on sagittal T1-weighted images. Slice locations are, -20, -46 mm (MNI) from left to right.

However, the t-score strictly requires a Gaussian distribution in order for FA values at each voxel to have the assumed theoretical t distribution with n-1 DF. Although the two-group t-test is known to be robust to deviation from the Gaussian distribution, this property cannot be applied to “one vs. many” t-test because when a patient is compared to a group of control subjects, the central limit theorem, which provides robustness in the two-group t-test, is no longer valid. One way to validate this Gaussian assumption is to compare theoretical variance and variance estimated from the data. The SD of the t-score can be estimated from the data simply by dividing the bootstrap SD estimate of the Z-score at each voxel by 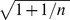. A comparison of the theoretical SD ( = 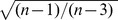) and bootstrap SD estimates of t-scores is demonstrated in Figure S1. The distribution of the bootstrap SD estimates of t-scores is approximately centered (Median = 1.05; Mean = 1.07) on the theoretical value 1.06 with n = 20, but widely spread, indicating deviation from the theoretical t distribution. Inferences that voxels are abnormal, based on the theoretical t distribution, may therefore be substantially biased for those voxels located at the tail regions of the histogram shown in Figure S1. Since about 60% of voxels showed smaller SD estimates than the theoretical SD, determination that voxels are abnormal based on “one vs. many” t-test tends to be conservative and subject to false negative inferences. Further, voxelwise bootstrap SD estimates were classified into 3 classes: (a) under-dispersion, (b) over-dispersion and (c) close to the theoretical SD value by comparing bootstrap SD estimates to the theoretical SD. Over-dispersion was frequently found in peripheral WM regions while under-dispersion was found in the deep white matter (Figure 3). Methods that apply a theoretical SD threshold uniformly across all voxels may thus produce higher false positive or false negative decisions for the over- and under- dispersion regions. Deviation from the theoretical variance suggests deviation from the Gaussian assumption for FA measures. For example, the distribution of FA at a voxel can be a mixture of two Gaussian distributions as in Equation (4).
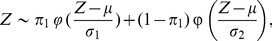
(4)with the standard Gaussian density φ, 0<π_1_<1, and σ_i_ >0 (i = 1, 2). The distribution of FA values in Equation (4) corresponds to a mixture of two subpopulations, each of which has Gaussian distribution with the same mean μ, but different variances. This model may be plausible in certain populations, for example, above a certain age, variance may increase or decrease significantly. Although, in the present study, effects of age on the mean FA were removed by linear regression, heterogeneous variance among age groups may remain. Under an assumption that the sample size (n) for the reference group is sufficiently large, the distribution of t-scores in Equation (3) derived with samples from a mixture Gaussian distribution is found to be a mixture of two t-distributions. Accordingly, classification of a voxel as abnormal based on the theoretical t-distribution is not valid; the coverage rate bounds (- t_n-1,α/2_, t_n-1,α/2_) from the theoretical t distribution cannot achieve the desired coverage rate [(1-α)×100%] in the presence of a mixed Gaussian distribution as in Equation (4). Details of this derivation are provided in Text S2.

## Results

**Figure 4:**
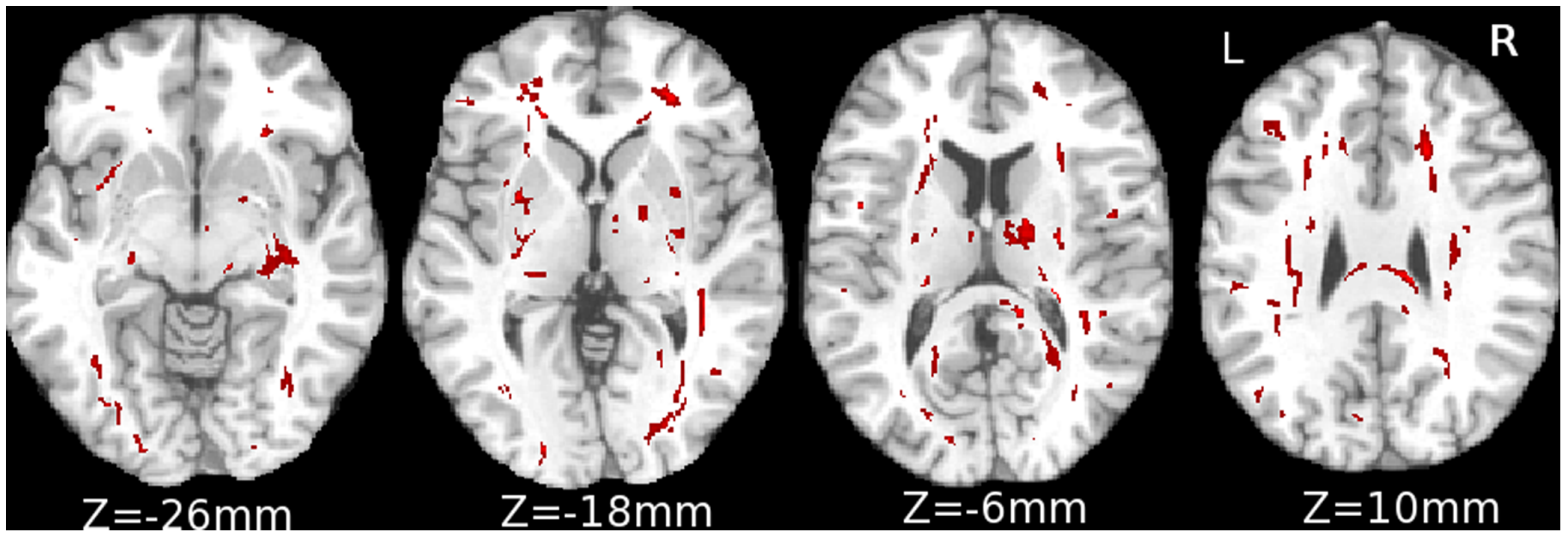
White matter regions with significant effect of demographic variables. White matter regions demonstrating significant multiple regression effects with three covariates (age, gender, years of education) (F (0.05, 3, 17) = 3.2 and cluster size over 100 voxels). Example images are at Z = −26, −18, −6 and 10 mm (Montreal Neurological Institute (MNI) coordinates).

Characteristics of the patient and control samples (Table 2) show that the range of age, gender, and years of education of controls fully encompasses those of patients. Furthermore, no significant difference in age (t-test; p = 0.289, 0.324) or gender distribution (Chi-squared test; p = 0.864, 0.511) was found between each control group and the patient group. However, a significant difference in years of education (t-test; p = 0.005, 0.004) was found. 27,290 white matter voxels (4.5% of total white matter volume) met significance criteria for the effects of demographic covariates, dominated by the effect of education. Four axial slices with significant effects of demographic covariates on FA are shown in Figure 4.

**Figure 5:**
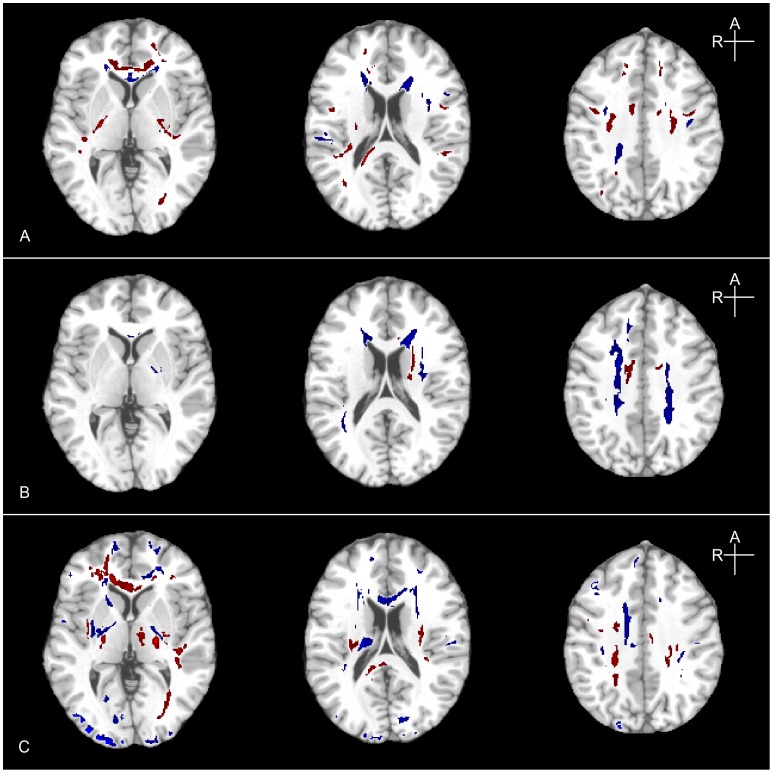
Variation in the spatial distribution of FA abnormalities across patients. Three axial images in three patients (A, B, C) showing multiple areas of abnormally high (blue) and low (red) FA in the acute post-injury period (A- 3days, B- 6days, C-9days). Each patient shows multiple locations of abnormality, with variable lesion location across individuals.

Figure 5 shows example maps of abnormal FA from three different mTBI patients, demonstrating multiple areas of abnormally high and low FA with significant variation in the size and spatial distribution of FA abnormalities across patients. The determination of abnormality at this stage was defined using thresholds (α_1_ = 0.05 (2-tails); α_2_ = 0.05 (corrected for multiple comparisons)) determined from the ROC analysis (below).

In assessing diagnostic utility in individual patients, we initially investigated three global metrics: (1) all abnormal white matter voxels across the whole brain, (2) all white matter voxels where FA was significantly lower than normal and (3) all white matter voxels where FA was significantly higher than normal. Table 3 shows AUC and p-values (Wilcox-Mann-Whitney test, 1-tailed) calculated at various levels of the two thresholds, α_1_ and α_2,_ for the first global metric (all abnormal white matter voxels across the whole brain). Greatest efficacy in making the discrimination between patients and controls, based on maximizing AUC, was found at lower thresholds for α_1_ and higher thresholds for α_2._

**Table 3:** Comparison of thresholds (α_1_, α_2_) based on AUC.

Cluster size threshold (α_2_)				
Individual voxel threshold (α_1_)	5% uncorrected	1% uncorrected	5% corrected	1% corrected
**(α_1_ = 5%)**				
EZ-MAP	0.701 (0.002)	0.701 (0.002)	0.724 (0.000)	0.702 (0.002)
“1 vs. many” T test	0.702 (0.002)	0.710 (0.001)	0.738 (0.000)	0.718 (0.001)
Z-score	0.689 (0.004)	0.692 (0.003)	0.703 (0.002)	0.696 (0.003)
**(α_1_ = 1%)**				
EZ-MAP	0.678 (0.007)	0.668 (0.011)	0.695 (0.003)	0.702 (0.002)
“1 vs. many” T test	0.665 (0.012)	0.678 (0.007)	0.705 (0.002)	0.695 (0.003)
Z-score	0.678 (0.007)	0.674 (0.008)	0.685 (0.005)	0.681 (0.006)

Note – Discrimination between patients and controls based on a global metric, (all abnormal white matter voxels across the whole brain) assessed by AUC score. AUC is tested by Wilcox-Mann-Whitney test (1-tailed); AUC score and its p-value in parentheses are calculated for each pair of two thresholds.

Similarly, ROC studies were conducted for the “one vs. many” t-test and the standard Z-approach to find optimal thresholds; these results are summarized in Table 3. All three methods showed maximal AUC scores at α_1_ = 0.05 (2-tails); α_2_ = 0.05 (corrected for multiple comparisons). This pattern is opposite to that for FWER control, where thresholds less strict for α_1_ but fairly strict for α_2_ yield optimal discrimination power. Table 4 shows the sensitivity and specificity achieved when applying optimized thresholds (α_1_ and α_2_) for each method.

**Table 4:** Sensitivity and specificity from the ROC analysis.

	EZ-MAP	“1-vs.-many”T test	Z-score	FWER- control
**Sensitivity**	0.706	0.647	0.647	NA
**Specificity**	0.714	0.762	0.762	NA

Note- EZ-MAP, “one vs. many” T, and Z-scores were thresholded at α_1_ = 0.05 (2-tails); α_2_ = 0.05 (corrected for multiple comparisons). In the ROC analysis, the optimal cut-off number (all abnormal voxels for discrimination of patients and controls) is the point closest to the top-left corner of the ROC curve. Sensitivity and specificity are not applicable because the FWER-control method was not significantly powered in the ROC analysis.

**Figure 6:**
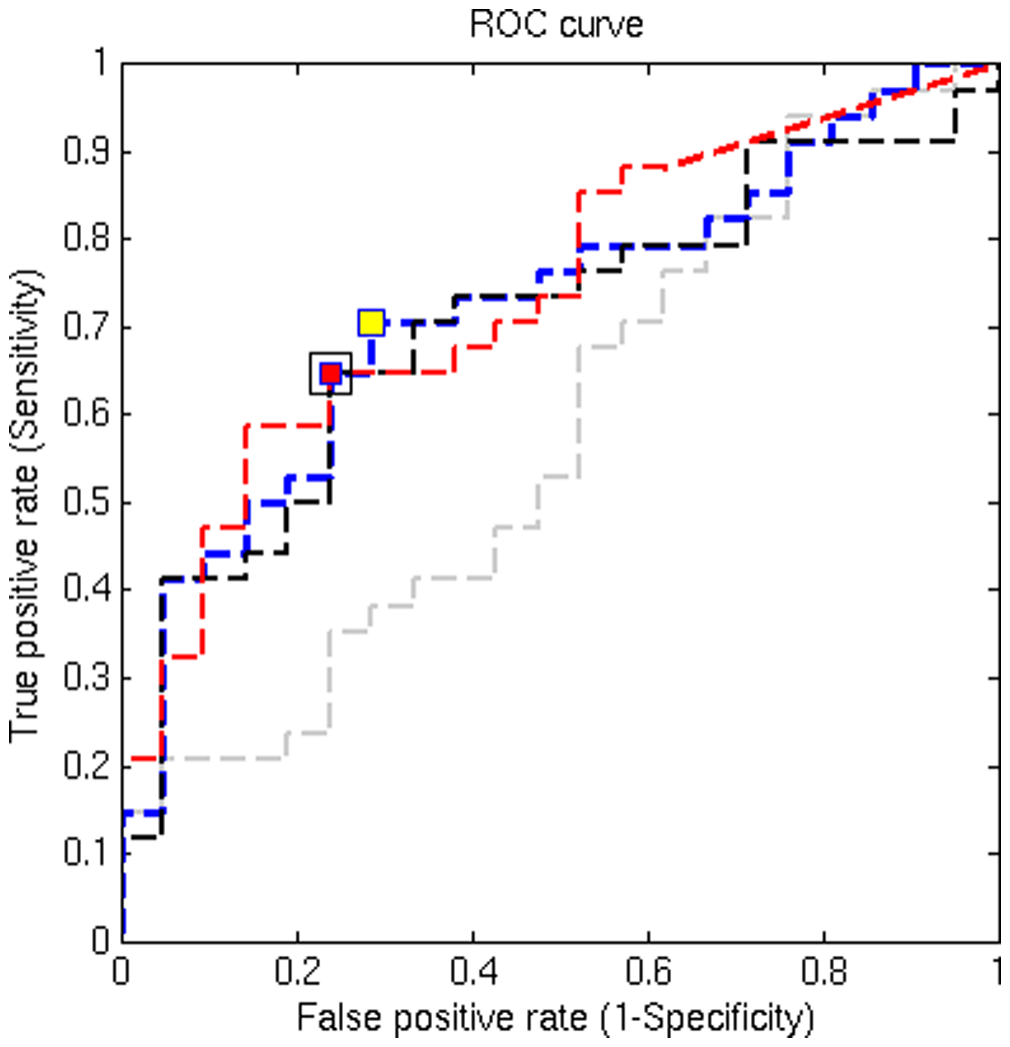
ROC curves from four analytic methods. ROC curves from four analytic methods, (1) EZ-MAP, (2) “one vs. many” t-test, and (3) standard Z-score approach and (4) the FWER test with pseudo t-distribution, were compared for one global metric (number of whole brain white matter voxels meeting criteria for abnormality). The thresholds applied were α_1_ = 0.05 (2-tailed) and α_2_ = 0.05 (corrected for multiple comparisons) for the EZ approach, “one vs. many” t-test, and standard Z-score approach while α_1_ = 0.05 (1-tailed) with no spatial extent threshold was employed for the FWER test. The ROC curve for each of the three analytical methods is shown (blue dotted line = EZ-approach, red dotted line = “one vs. many” t-test, black dotted line = standard Z-score, grey dotted line = FWER test). The EZ-MAP, “one vs. many” t-test, and standard Z-score approaches showed significant power; the yellow square indicates the optimal sensitivity and specificity of the ROC curve for EZ-MAP (sensitivity- 71%, specificity 71%); red and black squares indicate this point for the “one vs. many” t-test and standard Z-score, respectively (sensitivity 65%, specificity 76% for both methods). The FWER test was not significant and therefore optimal sensitivity and specificity are not noted.

**Figure 7:**
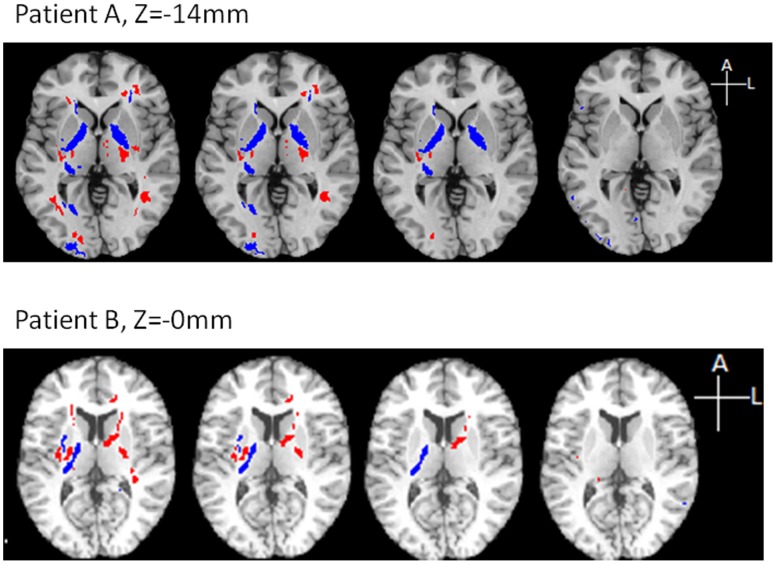
Abnormal regions detected by four analytic methods. Two example axial slices (z = −14 and −0 mm in MNI coordinates, from top to bottom) for detected abnormalities in two different mTBI patients are presented. Analysis methods are arranged as, from left, standard Z-score, EZ-MAP, “one vs. many” t-test and FWER-control. Abnormal regions are colored blue (abnormally high) and red (abnormally low). While similar locations are classified as abnormal by the various methods, fewer of the abnormal regions survive among analysis methods from left to right.

**Figure 8:**
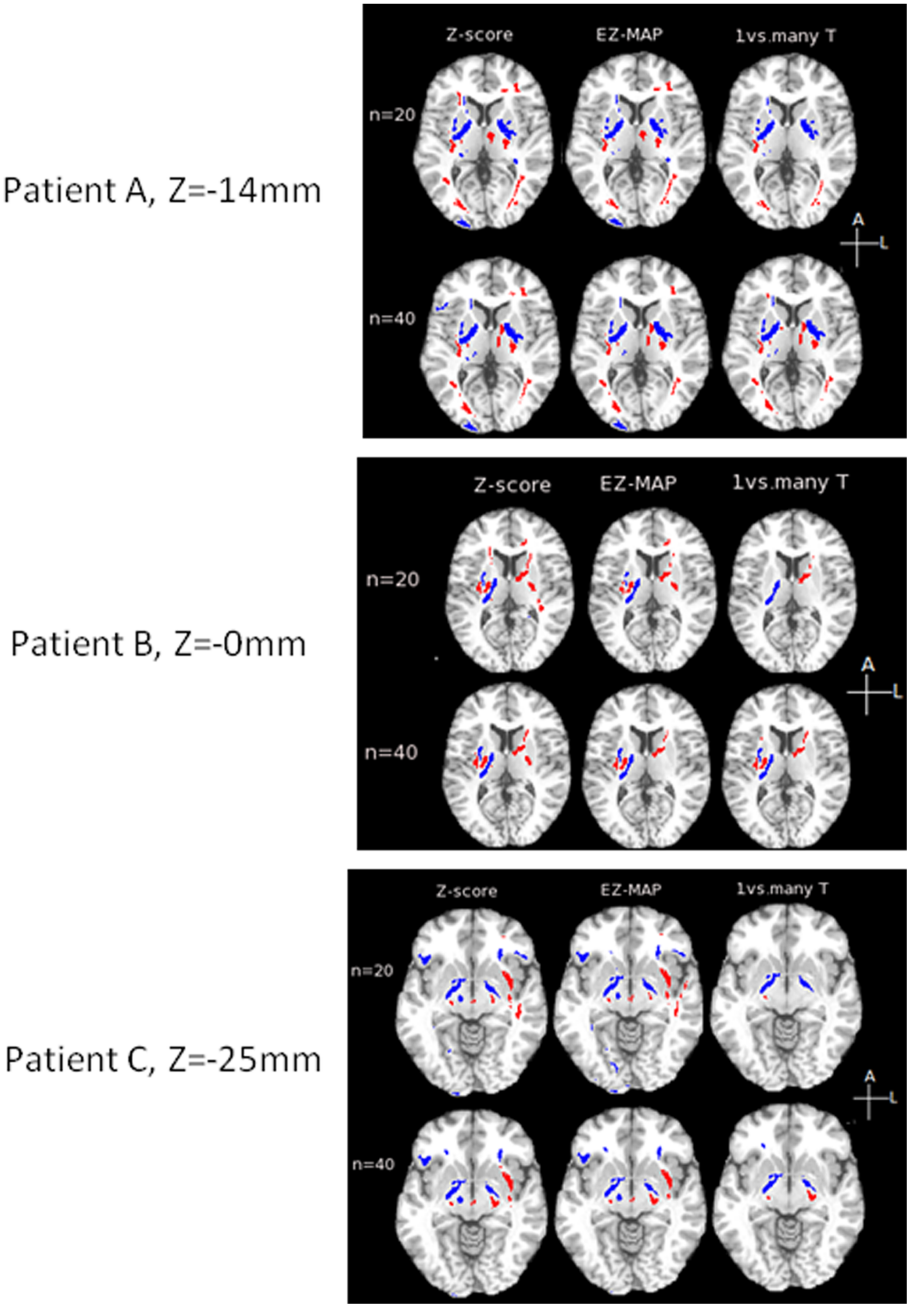
Variability in the abnormality maps by changing the size of the reference group. Regions classified as abnormal are shown, based on two reference groups (top n = 20; bottom n = 40) with 3 axial slices for each of 3 different mTBI patrients (A, B, C). In each row, the standard Z-score result is shown at left, EZ-MAP in the middle and “one vs. many” t-test on the right. Abnormal regions are colored blue (abnormally high) and red (abnormally low).

Diagnostic utility of the different analysis methods were compared for each of the three global metrics derived at the optimized thresholds [(α_1_ = 0.05 (2-tails); α_2_ = 0.05 (corrected for multiple comparisons) for EZ-MAP, standard Z-score and one vs. many t-test, while FWER-control was tested at 5% for each tail area]. Overall, all of the three global metrics attained significant power to discriminate mTBI patients from controls using EZ-MAP, standard Z-score or “one vs. many” t-test. However, the discrimination power of the FWER control approach was not significant as shown in Table 5 and Figure 6. The first global metric (all abnormal FA voxels) was somewhat more significantly different between groups than the other two metrics (all abnormally low FA voxels or all abnormally high FA voxels). Two example axial slices from two different patients are presented in Figure 7. As shown in Table 5 and Figure 7, the standard Z-score approach identifies the largest number of voxels, while EZ-MAP identifies fewer and the “one vs. many” T-test still fewer abnormalities. The FWER-control identified the fewest voxels as abnormal. Interestingly, the number of abnormal voxels from standard Z-score was about 1.5 times greater than the number from EZMAP, and again the number from EZ-MAP was about 1.5 times greater than the number from “one vs. many” t-test. Abnormal regions detected by the standard Z-score should contain more false positives due to underestimation of variance in comparison to EZ-MAP. Since 60% of voxels showed under-dispersion when compared to the expected theoretical variance of the t-distribution, the number of voxels classified as abnormal by the “one vs. many” t-test is excessively conservative. The FWER-control approach is also overly conservative. Robustness of the spatial extent of abnormalities for individual patients derived with different control subjects were explored, as demonstrated in Table 6 and Figure 8. Two sets of reference groups (n = 20 and n = 40; the n = 40 group includes the n = 20 group plus an additional 20 control subjects) were used separately to assess individual mTBI patients. For each of the three global metrics the number of abnormal voxels detected by standard Z-score approach greatly decreased as the size of the reference group increased, while “one vs. many” T-test showed the opposite pattern. The regions of abnormally low FA detected with the EZ-score were stable across reference group. Regions of abnormally high FA detected with the EZ-score, however, decreased as the size of the reference group increased.

**Table 5:** Assessment of discriminatory ability.

Number of abnormalwhite matter voxels	Mean and SD of the numbers of abnormal voxels across subjects		Significance of group difference (p-value)	
	Normal Subjects (n = 21)	mTBI Patients (n = 34)	2-group T	W-M
**All Abnormal Voxels**				
EZ-MAP	6211 (6282)	14000(11108)	0.003	0.000
“1 vs. many” T test	3147 (4356)	8759 (8206)	0.003	0.000
Z-score	11783(9393)	21603(14459)	0.004	0.002
FWER-control	1136 (625)	1459 (1092)	0.112	0.164
**Abnormally Low Voxels**				
EZ-MAP	2285(3189)	6489(7800)	0.012	0.077
“1 vs. many” T test	1058(2255)	4134(6559)	0.022	0.063
Z-score	4315(4977)	9998(10661)	0.013	0.042
FWER-control	756 (494)	941(753)	0.161	0.253
**Abnormally High Voxels**				
EZ-MAP	3925(4463)	7512(6850)	0.019	0.007
“1 vs. many” T test	2089(2796)	4625(4763)	0.016	0.017
Z-score	7468(6845)	11605(9232)	0.041	0.046
FWER-control	380 (539)	518 (770)	0.478	0.184

Note- Mean number of abnormal voxels and SD (in parentheses) detected by each analysis method from each group for each global metric (1) “All” - all abnormal white matter voxels across the whole brain, (2) “Low” - all white matter voxels where FA was significantly higher than normal and (3) “High” - all white matter voxels. Two-group t-test and Wilcox-Mann-Whitney test was conducted for each global metric to compare the numbers of abnormal voxels between normal subjects and mTBI patients.

**Table 6:** Robustness: Number of abnormal voxels (n = 34).

	Tested with n = 40 Control subjects	Tested with n = 20 Control subjects	Paired T-test (p-value)
**All abnormal Voxels**			
EZ-MAP	12965 (11623)	14000 (11108)	0.725
“1 vs. many” T test	10271 (10534)	8759 (8206)	0.537
Z-score	16516 (13383)	21603 (14459)	0.156
**Abnormally Low FA Voxels**			
EZ-MAP	6400 (8314)	6489 (7800)	0.965
“1 vs. many” T test	5187 (7641)	4134 (6559)	0.566
Z-score	8129 (9657)	9998 (10661)	0.475
**Abnormally High FA Voxels**			
EZ-MAP	6565 (6548)	7512 (6850)	0.590
“1 vs. many” T test	5083 (5464)	4625 (4763)	0.736
Z-score	8387 (7738)	11605 (9232)	0.149

Note- Mean number of abnormal voxels (SD) detected by each analysis method from each group for each global metric (1) “All” - all abnormal white matter voxels across the whole brain, (2) “Low” - all white matter voxels where FA was significantly higher than normal and (3) “High” - all white matter voxels. Paired t-test was conducted for each global metric to compare numbers of abnormal voxels detected in mTBI patients using each of two reference groups (n = 20, n = 40).

## Discussion

Individualized assessments are needed to guide personalized therapeutic interventions [25]–[30]. Personalized medicine is generally understood to encompass genotype-tailored treatment [25]–[29], but other unique manifestations of disease demand individualized diagnostic and therapeutic approaches. Individualized assessment of DTI has been reported in only a few studies of TBI, which applied group-wise methods to individuals [4], [6], [8], [31]. However, individualized assessments are especially relevant to TBI, where the nature of the injury and its pathologic manifestations will be unique in each individual [10]. Additionally, the analytic methods we have described could, after appropriate validation, be generalized to the assessment of many brain diseases.

We carefully addressed several important considerations in the implementation and validation of our approach. First, any study must in practice employ a control group that is a small subset of the population against which determinations of abnormality are to be inferred. This sampling limitation may lead to underestimation of variance in Z-scores and consequent erroneous inferences. EZ-MAP accounts for this potential additional variance by bootstrap, a nonparametric method which resamples the Z-score (i.e., by resampling the composition of the control group). An alternative bootstrap method would first resample the deviation of an individual FA from the mean FA obtained from a control group arriving at a new bootstrap SD quantity, which would then be used in computing the individual patient Z-scores. We employed the former approach because it yields a more robust and stable approximation of the true distribution [32].

We did not incorporate bias (variability of the control group mean based on its composition) in calculating the EZ-MAP, assuming that bias would be very close to zero. The narrow distribution (particularly around zero) of the mean of resampled Z-scores at each voxel confirms the validity of this assumption (Figure S2).

We further confirmed that differences between the patient and control groups were carefully assessed and accounted for to minimize the chance that covariates such as age, gender and education would be detected as real effects. Notably, we found that significant effects of age, gender and education were modest in magnitude and spatial extent.

EZ-MAP discriminated mTBI patients from normals, showing statistical significance on assessment of area under the ROC curve and significant differences between patients and controls in the number of abnormal voxels detected. In terms of discrimination power, the standard Z-score and “one vs. many” t-test approaches also attained significance while an approach employing FWER-control did not. Although all three methods achieved significant discrimination power, the extent of abnormal regions varied among the methods. Inferences based on the standard Z-score approach tend to produce more false positive findings and those identified with the “one vs. many” t-test yield more false negative inferences. EZ-MAP inferences fall in between these two extremes. Because EZ-MAP is a data-adaptive approach, it is inherently less sensitive to underlying assumptions regarding the composition of the reference group than standard Z-score approach and “one vs. many” t-test.

Several potential limitations of our study should be considered. The assumption that the distribution of FA at each voxel would be symmetric, implicit in the standard Z-score, EZ-MAP and the “one vs. many” t-test, was explored because we suspected that such assumptions would lead to erroneous inferences. The density functions of EZ-scores from each normal control subject (Figure S3, top) and all subjects (Figure S3, bottom), were estimated by mixture modeling [33]. In the estimated density functions for individuals, we found a thicker tail to the right (highly positive EZ-scores). The estimated density function for all control subjects also showed a thicker tail to the right in comparison to the standard Gaussian distribution. As a consequence of this asymmetry, abnormally high FA voxels (i.e., the right tail) were likely to be identified in “normal control subjects”. Such “abnormalities” might be detected due to deviation from underlying assumptions about the control group (i.e., the absence of a symmetric distribution across subjects at each voxel) not due to actual abnormalities. Therefore, a further improvement for analysis of DTI images in individual patients beyond the EZ-MAP may be achieved by developing methods to account for potential asymmetry in the FA distribution.

It is also important to critically assess the likelihood that the effects seen in the mTBI patients we studied are due to mTBI rather than some other white matter abnormality. Although, strictly speaking, it would never be possible to accept a null hypothesis that our patient and control subjects do not differ other than due to mTBI, we went to great lengths to ensure, to the maximum extent possible, that differences between patients and controls are reasonably attributable to mTBI. The mTBI patients enrolled in this study were carefully screened to exclude pre-injury medical, neurological or psychiatric disorders, including substance use, which could possibly cause white matter pathology. In addition to adjusting for age, we excluded patients at extremes of the lifespan, where developmental or senescent changes may affect FA. Although a significant difference in education was found between patients and controls, we found only minimal effects on FA, which were adjusted for in our analyses. Finally, abnormal findings occurred in areas expected to be affected in TBI (e.g., [1], [3]–[10], [31], [34]–[45]) and are consistent with numerous prior studies of DTI in TBI (e.g., [4], [6]–[8], [36], [38]–[40]).

## Supporting Information

Figure S1 **Bootstrap SD estimates of T-scores.** Bootstrap SD estimates of t-scores from all white matter voxels across the whole brain is compared to the theoretical SD ( = 1.06) from the t-distribution with DF = 19. The thin red bar indicates the theoretical SD. About 60% of the voxels are located below the theoretical SD.
(TIF)

Figure S2 **Histogram of the mean of resampled Z-scores.** Histogram of the mean of resampled Z-scores at each voxel, based on the bootstrap procedure (Text S1) is shown. The histogram is approximately centered at zero with a narrow width (±0.1).
(TIF)

Figure S3 **Estimated density function of EZ-scores.** The density function of EZ-scores was estimated for each normal control subject (top, blue) and all subjects (top, red), by concatenating individual volumes. The estimated density function for all control subjects (bottom, red) was compared to the standard Gaussian density function (bottom, black).
(TIF)

Text S1 **Bootstrap procedure for estimation of the SD for use in Enhanced Z-scores.**
(DOC)

Text S2 **Derivation of the distribution of T-score with random samples from a mixture of two Gaussian distributions.**
(DOC)
