# Supplementary figures and images for: Whole Brain Approaches for Identification of Microstructural Abnormalities in Individual Patients: Comparison of Techniques Applied to Mild Traumatic Brain Injury
Source: PLoS One. 2013 Mar 26;8(3):e59382. doi: 10.1371/journal.pone.0059382 (PMC3608654; doi:10.1371/journal.pone.0059382)

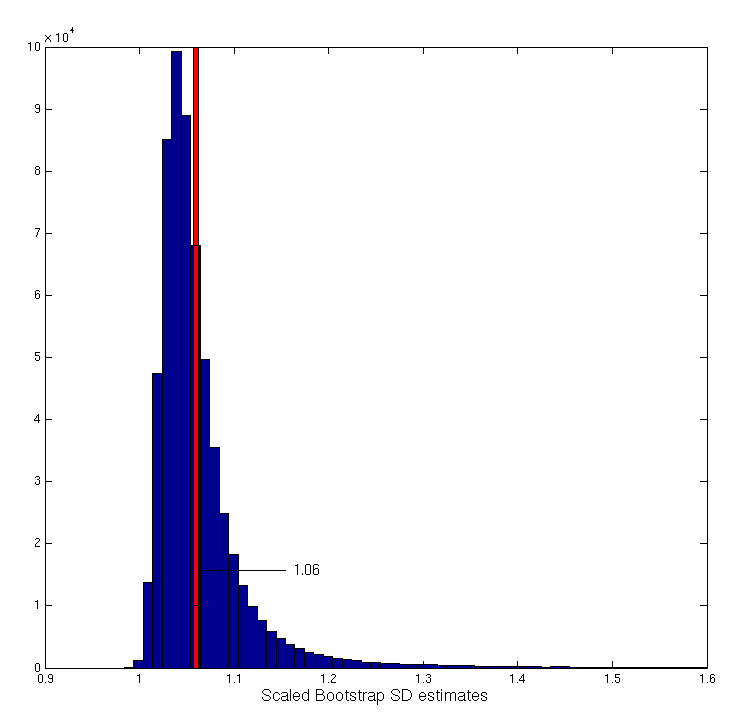

Supplement: Figure S1 — Bootstrap SD estimates of T-scores. Bootstrap SD estimates of t-scores from all white matter voxels across the whole brain is compared to the theoretical SD ( = 1.06) from the t-distribution with DF = 19. The thin red bar indicates the theoretical SD. About 60% of the voxels are located below the theoretical SD. (TIF) [file pone.0059382.s001.tif]

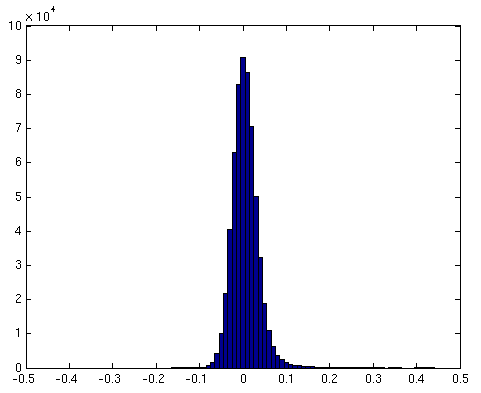

Supplement: Figure S2 — Histogram of the mean of resampled Z-scores. Histogram of the mean of resampled Z-scores at each voxel, based on the bootstrap procedure (Text S1) is shown. The histogram is approximately centered at zero with a narrow width (±0.1). (TIF) [file pone.0059382.s002.tif]

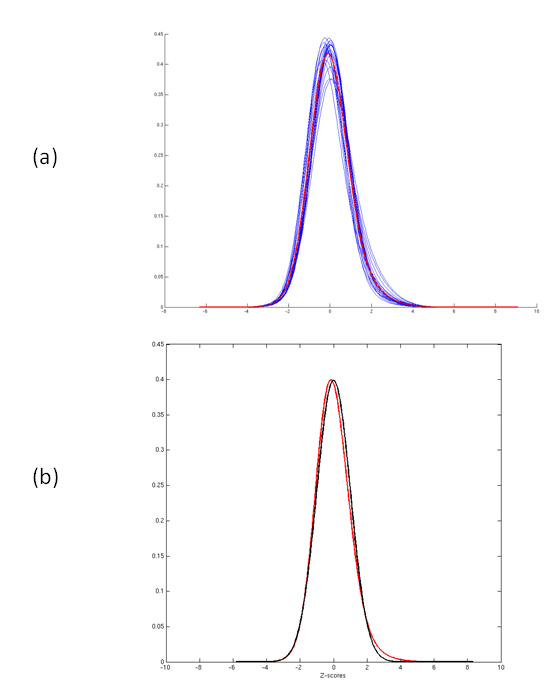

Supplement: Figure S3 — Estimated density function of EZ-scores. The density function of EZ-scores was estimated for each normal control subject (top, blue) and all subjects (top, red), by concatenating individual volumes. The estimated density function for all control subjects (bottom, red) was compared to the standard Gaussian density function (bottom, black). (TIF) [file pone.0059382.s003.tif]
