# Supplementary material for: Whole Brain Approaches for Identification of Microstructural Abnormalities in Individual Patients: Comparison of Techniques Applied to Mild Traumatic Brain Injury
Source: PLoS One. 2013 Mar 26;8(3):e59382. doi: 10.1371/journal.pone.0059382 (PMC3608654; doi:10.1371/journal.pone.0059382)
Supplement: Text S1 — Bootstrap procedure for estimation of the SD for use in Enhanced Z-scores. (DOC) [file pone.0059382.s004.doc]

**Text S1.** **Bootstrap procedure for estimation of the SD for use in Enhanced Z-scores**

(n+1) FA values of control subjects

Repeat B times (b=1,…,B)

b^th^ bootstrap sample:

,

Where $\bar{x}_{\sim k^{*}}^{(b)}$ and $s_{\sim k^{*}}^{(b)}$are mean and SD of b^th^ bootstrap sample respectively, and k^*^ is an integer predetermined (k^*^ = n+1 for this study).$\bar{z}^{B}$is the mean of $z^{(b)}$(b=1,…,B) with B=2000.
