# Supplementary material for: Whole Brain Approaches for Identification of Microstructural Abnormalities in Individual Patients: Comparison of Techniques Applied to Mild Traumatic Brain Injury
Source: PLoS One. 2013 Mar 26;8(3):e59382. doi: 10.1371/journal.pone.0059382 (PMC3608654; doi:10.1371/journal.pone.0059382)
Supplement: Text S2 — Derivation of the distribution of T-score with random samples from a mixture of two Gaussian distributions. (DOC) [file pone.0059382.s005.doc]

**Text S2. Derivation of the distribution of t-score with random samples from a mixture of Gaussian distribution**

(x_1_,…,x_n+1_) is denoted as random samples from a mixture of two Gaussian distribution as in Equation (A.1), and t-score for x_n+1_ is derived from Equation (3).

, (A.1)

where σ_1_=1 for simplicity from Equation (4).

T-score can be written as a weighted sum of two T random variables as in Equation (A.2), if n is sufficiently large that $\bar{X}_{n}$ approximates to a Gaussian distribution.

, (A.2)

where $\bar{X}_{n}$is the mean of X_1_,…,X_n_, , , , , and .

Due to deviation from the theoretical T-distribution, t(n-1) in this example, abnormality decision based on the theoretical distribution is not accurate. For example, we may apply (1-α)×100% C.I. for abnormality decision from the assumed T-distribution, which is ±t_n-1,α/2_. However, the true coverage rate from the underlying mixture distribution is . The coverage rate achieves (1- α) if π_1_=1or 0, or σ=1, which brings back to a Gaussian distribution for all X_i_’s.
